# Chronic Chloral Poisoning

**Published:** 1878-11

**Authors:** Frank Woodbury


### CHRONIC CHLORAL POISONING.

BY FRANK WOODBURY, M.D.

Since 1869, when it was brought to the notice of the profession by Liebreich, choral rapidly gained general favor. Standing as it does in the front rank of cerebral spinal sedatives, it is now almost universally employed, being, according to Fothergill, in conditions of sleeplessness due to vascular excitement, "the hypnotic *par excellence*," and is "far superior to opium." This and the other advantages of the internal administration of the drug, in acute mania, in convulsions, whether puerperal, hysterical, epileptic, or infantile, and most markedly in tetanus, and in alleviating the paroxysms of hydrophobia, its use in whooping cough and other nervous affections, having been frequently referred to in our discussions, are sufficiently known, and are familiar as household words. I would, however, ask the indulgence of this Society, while I invite its attention to some peculiar effects that have been observed after the long continued administration of this drug, in what has been considered ordinary medicinal doses. From the fact that death has occurred immediately after the administration of single large doses, and a sufficient number of such cases has been recorded to establish this fact, we are justified in making the assertion that chloral hydrate is capable, when introduced into the system, of causing death, as a direct effect of its administration. It is, however, to the effects of chloral short of the fatal result, from doses sufficient to produce decided toxic symptoms without determining the final cessation of the vital functions, that our attention may, perhaps not unprofitably, be directed this evening.

As to the fatal dose and its relation to the ordinary dose of chloral, there is a wide latitude observed in the experience and opinion of different individuals. Dr. Richardson, of London, says that 180 grains is a fatal dose, and that it would not be safe to give more than 120 grains in 24 hours, as the system cannot decompose and eliminate more than five or seven grains per hour. On the other hand, in the experience of members of this Society, this has been largely exceeded, not only without a bad result, but apparently with great benefit to the patient. And yet Dr. Fuller (*Lancet*, March, 1871) reports a case of death following the administration of 30 grains in a healthy young lady; and Dr. Schwaighofer, of Vienna, records (*Irish Hospital Gazette*, 1873) a similar result from the same dose, in a drunkard. Dr. E. F. Ingalls (Chicago *Medical Journal and Examiner*) reports a case of death in a healthy German woman following the administration of ten grains of the remedy, a similar dose having been administered an hour before without ill effects, and no other medicine having been given.

How can this be reconciled with the fact that more than once, in the treatment of delirium tremens, one full ounce has been given in the twenty-four hours, not only without danger, but with the best results?

Now, in these cases, the fault lies either with the drug or the individual. The statement may be made that an impure article had been used in the fatal cases, and that some of the secondary products of decomposition, being more noxious than the chloral itself, had caused death. The justice of this observation is acknowledged, and it may be accepted as a sufficient explanation for some of the cases, but others yet remain in which the same article had been given to other patients with only the ordinary effects; and we can safely assume that, as a rule, in practice every ordinary precaution is undoubtedly taken to obtain the medicine in a state of integrity. Moreover, in Europe it has been commonly prescribed in much larger doses, and almost uniformly without bad results. We have all met with paticnts who cannot take opium. In many cases there is, probably in like manner, a chloral idiosyncracy, either natural or acquired; the latter class including persons suffering from such disorder of the nervous system, or heart, as renders them peculiarly susceptible to the poisonous action of the drug. Fothergill points out the fact that in an anæmic condition of the nervous centres chloral is contra-indicated; and DaCosta advises caution in its administration, and remarks that "in cases of cardiac debility, and in dilation, or much obstruction of the heart, it is generally contra-indicated. (DaCosta, Clinical Notes on Chloral, *American Journal of Medical Science*, April, 1870.)

Without lingering upon this interesting question of the causes of such idiosyncrasy, it will doubtless be conceded that individual peculiarities render certain persons peculiarly susceptible to chloral, so that in a large number of patients we may reasonably expect to find a few in which unusual and alarming symptoms may follow the ordinary dose, as in the cases reported by Dr. Ingalls and Dr. Fuller. This is so well established that Dr. Wood (Therapeutics and Materia Medica, Second Edtion, Phila., 1877, p. 326) says: "I think the practical deduction from these facts is that twenty grains is the highest safe dose of the remedy, that this dose should not be repeated oftener than once an hour, and after sixty grains has been taken, not for some hours, unless in very urgent cases."

I have been the more particular in establishing the ordinary medical dose of chloral because, in many of the cases reported, where this drug has been given for a length of time, it is merely stated that it was given in the usual dose. I have had under my notice several cases where the effects of chloral in these generally considered safe doses have been so much liked by the patients that they continued taking the prescription for months on their own responsibility. These cases, however, when in the male sex are so generally associated with an indulgence in alcoholic stimulants, as to render it a matter of uncertainty whether the nervous symptoms were produced by the chloral or the alcohol. In the following case, under the care of Dr. DaCosta, it was clearly shown that delirium tremens was directly produced by the chloral, no alcohol having been taken for several months previously.

Mr. A., thirty-five years of age, American, a man of fortune and indulgent in his habits, had been always a free liver. Without preparation he was induced to absolutely resign all alcoholic stimulants. Shortly afterward he sought medical advice for sleeplessness and nervousness. He was ordered chloral, and found it very soothing in doses of twenty or thirty grains, at night. Being pleased with the effect of the prescription he discontinued his visits to Dr. DaCosta, and of his own accord had the medicine repeatedly renewed, gradually increasing the frequency and the amount of the dose, so that he constantly took from a drachm to a drachm and a half daily. He kept up this practice for several months, all the time being free from medical supervision. Although he was remonstrated with by several members of his family on this new indulgence, he considered the remedy not injurious to him, and as he liked the calming effects from it he could not be dissuaded from its use. After continuing the chloral thus for a period not far short of four months, he lessened the dose and then stopped, rather abruptly. The consequence was that his weakened nervous systom showed signs of great disturbance and an attack of characteristic delirium tremens supervened, with the wildest fancies and great sleeplessness. The pulse was feeble and moderately accelerated; the first sound of the heart was weak. There was general prostration of the muscular system, and much tendency to sweating. No odor of chloroform was detected in his breath, and of course, no alcohol. He complained of nausea and loss of appetite; his tongue was coated. One of the prominent features in the case was a disposition to leave his bed and walk about the room, while the muscular weakness was strikingly shown in the fact that he was very soon fatigued. However, it required a man to watch over him constantly to prevent his leaving the bed and trying to escape from his room. The case perfectly recovered, though slowly, under the use of small doses of morphine, a nourishing diet, and a moderate amount of alcoholic stimulants.

It is not my intention to make any comments upon this interesting case, but simply to present it before the Society in the hope of contrasting it with the experience of others. I may state, however, that a somewhat similar case of delirium tremens has been reported by Dr. Elliot, (*Lancet*, 1853, I, 754.)

On the other hand a member of this Society, Dr. Laurence Turnbull, in his recent work on "Anæsthesia," reports a case in which he "directed the employment of chloral hydrate in medicinal doses for one year, as a sedative and narcotic, and the only disagreeable result complained of by the patient was that it caused a hot feeling, with free perspiration, as if she were in a hot bath ; it was withdrawn at the end of that time without producing the least disturbance of the brain, inflammation of the skin, or loss of memory or intelligence," and he inclines to the opinion that "other causes besides the hydrate of chloral may have produced some of the recorded results."—*Medical and Surgical Reporter.*

